# Astrocyte galectin-9 potentiates microglial TNF secretion

**DOI:** 10.1186/s12974-014-0144-0

**Published:** 2014-08-27

**Authors:** Andrew J Steelman, Jianrong Li

**Affiliations:** Department of Veterinary Integrative Biosciences and Institute for Neuroscience, Texas A&M University, Mail Stop 4458, College Station, TX 77843 USA

**Keywords:** Galectin-9, TNFα, Astrocytes, Microglia activation, Poly(I:C), Neuroinflammation

## Abstract

**Background:**

Aberrant neuroinflammation is suspected to contribute to the pathogenesis of myriad neurological diseases. As such, determining the pathways that promote or inhibit glial activation is of interest. Activation of the surface glycoprotein T-cell immunoglobulin and mucin-domain containing protein 3 (Tim-3) by the lectin galectin-9 has been implicated in promoting innate immune cell activation by potentiating or synergizing toll-like receptor (TLR) signaling. In the present study we examined the role of the Tim-3/galectin-9 pathway in glial activation *in vitro*.

**Method:**

Primary monocultures of microglia or astrocytes, co-cultures containing microglia and astrocytes, and mixed glial cultures consisting of microglia, astrocytes and oligodendrocytes were stimulated with poly(I:C) or LPS, and galectin-9 up-regulation was determined. The effect of endogenous galectin-9 production on microglial activation was examined using cultures from wild-type and *Lgals9* null mice. The ability for recombinant galectin-9 to promote microglia activation was also assessed. Tim-3 expression on microglia and BV2 cells was examined by qPCR and flow cytometry and its necessity in transducing the galectin-9 signal was determined using a Tim-3 specific neutralizing antibody or recombinant soluble Tim-3.

**Result:**

Astrocytes potentiated TNF production from microglia following TLR stimulation. Poly(I:C) stimulation increased galectin-9 expression in microglia and microglial-derived factors promoted galectin-9 up-regulation in astrocytes. Astrocyte-derived galectin-9 in turn enhanced microglial TNF production. Similarly, recombinant galectin-9 enhanced poly(I:C)-induced microglial TNF and IL-6 production. Inhibition of Tim-3 did not alter TNF production in mixed glial cultures stimulated with poly(I:C).

**Conclusion:**

Galectin-9 functions as an astrocyte-microglia communication signal and promotes cytokine production from microglia in a Tim-3 independent manner. Activation of CNS galectin-9 likely modulates neuroinflammatory processes in which TNF and IL-6 contribute to either pathology or reparation.

**Electronic supplementary material:**

The online version of this article (doi:10.1186/s12974-014-0144-0) contains supplementary material, which is available to authorized users.

## Background

Reactive gliosis is associated with many neurodegenerative diseases and is considered a hallmark feature of neuroinflammatory diseases. It is well established that both microglia and astrocytes express various toll-like receptors (TLRs) [1], intracellular pathogen recognition receptors [2,3], and danger signal receptors and are capable of responding to their respective ligands, in part through the production of chemokines, cytokines and growth factors [4,5]. Given that dendritic cells are absent from the brain parenchyma and that the central nervous system (CNS) is devoid of a draining lymphatic system, it is believed that following infection of the CNS the initial recognition of invading pathogens by resident glia is paramount in facilitating the attraction, stimulation, and regulation of both the innate and adaptive arms of the peripheral immune system within this organ [6,7]. Moreover, mounting evidence suggests that inflammation in the CNS, which is orchestrated by reactive gliosis, ushers the reparation of damaged tissues, much as occurs in the periphery [8,9].

Because some products, in particular proinflammatory cytokines and reactive oxygen intermediates produced following glial activation, are potentially cytotoxic or result in intracellular stress, the magnitude, timing and duration of glial activation likely dictates the delicate balance between the beneficial and detrimental effects of neuroinflammatory responses. In humans, aberrant glial activation is associated with demyelinating diseases such as multiple sclerosis [10] as well as a broad spectrum of other neurological diseases including stroke [11], epilepsy [12–14], amyotrophic lateral sclerosis, Alzheimer’s and Parkinson’s diseases [15] and depression [16,17]. Likewise, in an animal model of multiple sclerosis, experimental autoimmune encephalomyelitis (EAE), microgliosis has been shown to contribute to disease onset and progression [18–20]. Furthermore, microglial production of the proinflammatory cytokines IL-6 and TNF appears to promote the development of seizures following experimental infection of the CNS with Theiler’s murine encephalomyelitis virus [21–25]. Acute glial activation following either peripheral or central administration of low-dose lipopolysaccharide (LPS) has been shown to induce symptoms of depression in mice [26–28]. Therefore, uncovering the factors that regulate glial activation is pertinent to understanding CNS disease pathogenesis and the return to homeostasis. Importantly, while much research has focused on the ability of either microglia or astrocytes to respond to inflammatory stimuli, far fewer studies have investigated potential intercellular pathways that are exploited between glia to orchestrate inflammatory responses within the CNS.

Galectins are β-galactoside-binding proteins that exhibit a broad range of functions, in part through their ability to bind branched glycans on glycoconjugates causing lattice formation and clustering of lipid rafts [29–31]. Recently, galectins have gained increased recognition for their ability to influence immune responses, either by acting as pathogen recognition receptors, modulating innate immune responses or by regulating adaptive immunity [32]. However, the functional role of galectin expression, especially galectin-9, within the CNS is incompletely understood. Studies *in vitro* have shown that galectin-9 can induce maturation of human monocyte-derived dendritic cells [33] and enhance proinflammatory cytokine secretion [34], which have been attributable to its ability to bind the highly glycosylated T-cell immunoglobulin and mucin domain-containing protein 3 (Tim-3) [34]. While galectin-9 protein or the gene encoding galectin-9, *Lgals9*, is not normally expressed at high levels within the CNS [35,36], it is increased following experimental pneumococcal meningitis [37] as well as infection with herpes simplex virus [38] or Japanese encephalitis virus [39] and is reportedly increased in the brains of multiple sclerosis patients [34,40], indicating that it becomes up-regulated during inflammatory conditions.

Herein, we report that astrocytes can promote microglial TNF production*.* Notably, recombinant galectin-9 potentiated both TNF and IL-6 secretion from microglia when concurrently administered with poly(I:C). Additionally, utilizing glia derived from *Lgals9*^*−/−*^ mice we found that galectin-9 was, in part, sufficient to elicit this effect. Together, these results indicate that CNS-derived galectin-9 can function as an interglial signaling molecule and may promote neuroinflammatory processes by enhancing microglial TNF production.

## Methods and materials

### Animals

Rat glial cultures were obtained from Sprague Dawley rat pups (P1-2; Harlan; Houston, TX, USA). Mouse cells were from wild-type (C57BL/6 J; The Jackson Laboratory, Bar Harbor, ME, USA), *tlr4* mutant (*tlr4*^*−/−*^; The Jackson Laboratory, Bar Harbor, ME, USA), *Lgals9* mutant (*Lgals9*^−/−^) and transgenic (Lgals9-EGFP) JF66Gsat/Mmucd mouse pups (P1-2). *Lgals9*^*−/−*^ mice were generated from heterozygous breeding pairs originally obtained from the Consortium of Functional Glycomics. Transgenic *Lgals9*:EGFP mice (Lgals9-EGFP) JF66Gsat/Mmucd strain were reconstituted from MMRRC. The experimental procedures described herein were approved by the Institutional Animal Care and Use Committee and were performed in accordance with guidelines of the National Institutes of Health.

### Glial cultures

Primary microglia, astrocytes and mixed glial cultures were isolated from the forebrains of Sprague Dawley rats or mice as described previously [36,41–43]. Briefly, brains were dissected and meninges removed, and the tissue was digested with HBSS containing 0.01% trypsin and 10 μg/ml DNase for 15 minutes at 37°C. The cells were then washed with DMEM containing 10% heat-inactivated FBS and 1% penicillin-streptomycin, filtered through a sterile 70-μm filter, then plated onto poly-d-lysine coated 75 cm^2^ flasks or directly into 24-well plates for experiments using mixed glia. The medium was changed every other day and cells were treated after seven to eight days *in vitro* (DIV). For experiments involving mono- and co-cultures, microglia were isolated by shaking the mixed glia-containing flasks for 1 hour at 200 rpm. After removing microglia, the flasks were shaken overnight to separate oligodendrocytes from the astrocyte layer. The resulting astrocyte cultures were treated with L-leucine methyl ester (1 mM) for 1 hour before subculturing to remove additional contaminating microglia. The purity of enriched monocultures was consistently greater than 94%. Co-cultures of astrocytes and microglia were reconstituted by plating microglia directly onto enriched astrocytes. The BV2 microglial cell line (a kind gift from Dr. Monica Carson, University of California, Riverside) was cultured in complete DMEM media consisting of DMEM supplemented with 10% FBS (Hyclone, Logan, UT, USA), L-glutamine (1 mM), sodium pyruvate (1 mM) (Sigma, St. Louis, MO, USA), penicillin and streptomycin (100 U each; Life Technologies, Carlsbad, CA, USA). Cells were treated with either poly (I:C) (0 to 50 μg/ml) or LPS (0 to 100 ng/ml) (Sigma, St. Louis, MO, USA) in DMEM medium supplemented with 0.1% BSA (Sigma, St. Louis, MO, USA) for 0 to 24 hours as indicated in the figure legend.

### Astrocyte dependent potentiation of microglial TNF production

**Figure 1:**
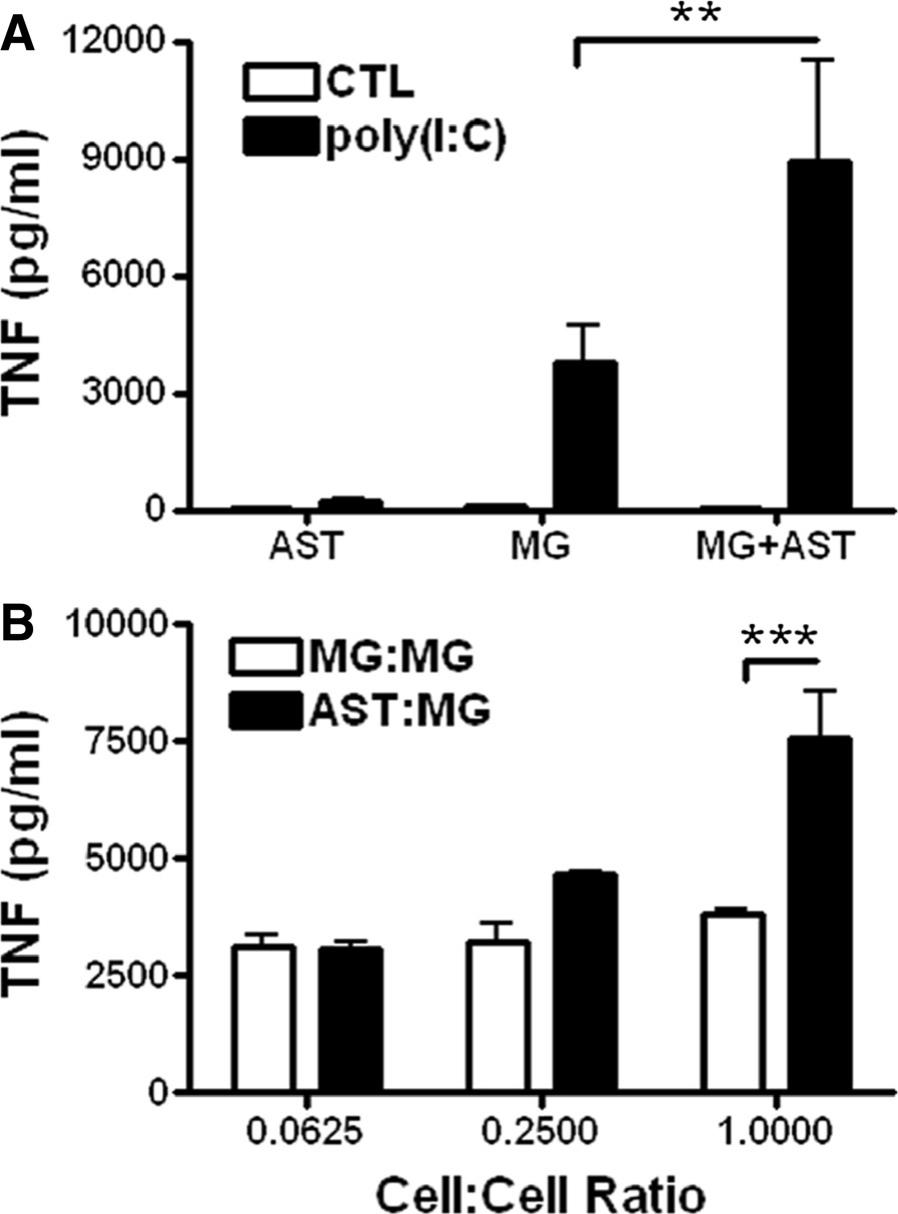
**Astrocytes enhance microglial TNF production after poly(I:C) stimulation. (A)** TNF levels from supernatants of mono- and co-cultures of microglia and astrocytes following stimulation with or without poly(I:C) (50 μg/ml) for 24 hours. Results are combined means ± SE of seven independent experiments. **(B)** Synergistic effect of astrocytes on microglial TNF is dependent on astrocyte number. Microglia were added in increasing numbers to microglia or astrocyte monocultures, and stimulated with poly(I:C) (50 μg/ml) for 24 hours and TNF measured from the supernatants. Results are combined means ± SE of three independent experiments. **P < 0.01, ***P < 0.001.

To determine the ability for astrocytes to promote microglial TNF production, we first employed a limiting dilution experiment. Here, microglia or astrocytes were plated into poly-d-lysine coated 96-well plates at a density of 5 × 10^4^ cells per well. After 24 hours, increasing numbers of newly isolated microglia were added to the above established monocultures to achieve cell ratios of 0.0625:1, 0.25:1 and 1:1 as described in the Figure 1 legend. The cells were then stimulated with poly(I:C) (50 μg/ml) for 24 hours and TNF measured in the supernatants by ELISA.

The necessity for living astrocytes to enhance TNF production from microglia was examined by culturing microglia (5 × 10^4^) alone or with astrocytes (5 × 10^4^) that had either been previously fixed with ice-cold methanol for 10 minutes or incubated with PBS and washed extensively. The cultures were incubated overnight at 37°C and then stimulated for 0, 4, 8 and 24 hours with poly(I:C). TNF production was then determined by ELISA.

To assess if the effect of astrocytes on microglia TNF production was contact-dependent, we compared the level of TNF production in microglia cultured with or without physical contact with astrocytes using methods described previously [41]. Briefly, 10 μl drops of Sylgard (polydimethylsiloxane; Dow Corning Corp., Midland, MI, USA) were spotted onto glass coverslips kept at low heat on a hot plate (Corning Inc, Corning, NY, USA) to create columns approximately 1 mm in height. The coverslips were sterilized, coated with poly-d-lysine, washed with sterile water and allowed to dry. Microglia were plated onto these ‘bridged’ coverslips, which were flipped over and placed into astrocyte cultures in 24-well plates as indicated to create non-contacting microglia/astrocytes co-cultures. For co-cultures with direct physical contacts between the two cell types, microglia were seeded directly into the astrocyte cultures. The cells were stimulated with poly(I:C) as described above for 24 hours.

### Immunocytochemistry

Cells were grown on poly-d-lysine coated glass coverslips in 24-well plates as described above. Following stimulation, the cells were fixed with 4% paraformaldehyde for 10 minutes at room temperature then washed with PBS. The coverslips were then blocked and permeabilized with PBS containing 0.1% Tween-20 (PBST) and 5% goat serum for 1 hour at room temperature (RT) then incubated with chicken anti-GFP (1/400; Life Technologies, Carlsbad, CA, USA), rat anti-GFAP (1/200; Millipore, Billerica, MA, USA) or rabbit anti-Iba-1 (1/2,000; Wako, Richmond, VA, USA) overnight at 4°C in PBST. Subsequently, the coverslips were washed 3× with PBS then incubated with fluorescently labeled goat anti-chicken, anti-rat or anti-rabbit IgG (1/1,000; Life Technologies, Carlsbad, CA, USA) for 1 hour in PBST containing 5% goat serum. After washing, the coverslips were mounted onto glass slides. Fluorescence intensity of GFP following stimulation with poly(I:C) was determined as described previously [44]. Images were captured using an Olympus DP70 digital camera mounted on an Olympus IX71 microscope at the same exposure time (Olympus, Tokyo, Japan). The background from each image was uniformly subtracted using Photoshop then the mean fluorescence intensity of each image was determined using ImageJ (NIH) [44]. Expression data were generated from four independent mixed glial cultures. For each time-point the mean fluorescence intensity of at least five random 10× fields were averaged per culture per time point. To determine microglial cell number in mixed cultures, Iba-1^+^ cells in at least five random fields were counted using ImageJ and averaged for each mouse. Averages from each mouse were used for statistical analysis.

### RNA isolation and RT-qPCR

RNA was extracted from cultured cells using Qiagen RNeasy kits according the manufacturer’s instructions (Qiagen, Valencia, CA, USA). Contaminating DNA was removed by digestion with DNase I (Life Technologies, Carlsbad, CA, USA). RNA was reverse transcribed using a Promega AMV kit (Promega, Madison, WI, USA). To test for residual DNA contamination, samples that were reverse transcribed in the absence of reverse transcriptase were examined for amplification following PCR. For non-quantitative RT-PCR, 100 ng cDNA was amplified by PCR. For quantitative PCR, 10 ng of cDNA was used for amplification. The specific primer pairs were used for amplification: rat Gal-9, Forward-GGCATACCCCACCCCAGCCT, Reverse-CAGGCAGGCTTCGCTCCTCG; rat GFAP, Forward-ACAAGGCGCTGGCAGCTGAG, Reverse-CACGTGGACCTGCTGCTGGG; rat Iba-1, Forward-CTTTTGGACTGCTGAAAGCC, Reverse-GTTTCTCCAGCATTCGCTTC; rat Tim-3, Forward-GTCCACATTGGAGTAGGCGT, Reverse-TGAGTGCAGTCTCTGGGTTG; β-actin, Forward-AGACTTCGAGCAGGAGATGG, Reverse-CCATCATGAAGTGTGACGTTG. For quantitative PCR, cDNA was amplified using SYBR® according to the manufacturer’s instructions (Life Technologies, Grand Island, NY, USA). All samples were run in duplicate or triplicate. Gene expression was normalized to β-actin and fold expression was calculated using the formula 2-^ΔΔCt^.

### Western blot

The expression of galectin-9 and GFP in mixed glial cultures following poly(I:C) stimulation was determined by western blot as described previously with slight modification [36,43]. Mixed glia were stimulated with poly(I:C) (50 μg/ml; Sigma, St. Louis, MO, USA) for 24 hours, lysed with Laemmli sample buffer, sonicated and frozen at −80°C until use. The lysates were separated by SDS-PAGE, transferred to PVDF membranes, blocked for 1 hour at room temperature and then incubated with goat anti-mouse Gal-9 (1/1,000; R&D Systems, Minneapolis, MN, USA), chicken anti-GFP (1/1,000; Life Technologies, Carlsbad, CA, USA) or mouse anti-β-actin (1/8,000; Sigma, St. Louis, MO, USA) overnight at 4°C. After washing, the membranes were incubated with horseradish peroxidase conjugated anti-goat IgG (1/2,500; Jackson ImmunoResearch, West Grove, PA, USA), anti-chicken IgY (1/5,000; Thermo Scientific, Logan, UT, USA) or anti-mouse IgG (1/30,000; Jackson ImmunoResearch, West Grove, PA, USA) for 1 hour at RT. Western blot were developed with Immun-Star WesternC reagent (Bio-Rad; Hercules, CA, USA) and images were acquired with a Bio-Rad Chemidoc XRS gel documentation system (Bio-Rad; Hercules, CA, USA) and quantified with Quantity One software (Bio-Rad; Hercules, CA, USA).

### Cytokine measurement from culture supernatants

Rat and mouse TNF was measured by ELISA according to the manufacturer’s instructions (eBioscience; San Diego, CA, USA). Mouse microglial TNF, IL-6, IL-1β, CCL2, IL-12(p40) and IL-10 secretion following recombinant mouse galectin-9 (2 μg/ml; R&D Systems, Minneapolis, MN, USA) and poly(I:C) stimulation were measured by Luminex according to the manufacturer’s instructions (Bio-Rad; Hercules, CA, USA).

### Flow cytometry

All antibodies used for flow cytometry were from eBioscience (San Diego, CA, USA). To determine Tim-3 surface expression, primary microglia or BV2 cells (1 × 10^5^) were suspended in 100 μl of flow cytometry buffer (PBS containing 2% FBS), blocked with anti-CD16/32 (clone 93) for 10 minutes on ice, stained with anti-CD11b-APC (clone M1/70), and anti-Tim-3-PE (clone RMT3-23) or an isotype control (IgG2a) for 20 minutes. To determine if exogenous galectin-9 could enhance microglial expression of MHC II and CD86 following poly(I:C) stimulation, cells (2 × 10^5^) were plated onto sterile 35-mm petri-dishes (Falcon; Becton Dickinson, Franklin Lakes, NJ, USA) and treated with recombinant galectin-9 (2 μg/ml) poly(I:C) (50 μg/ml), or both for 24 hours. The cells were detached with ice-cold Hanks’ Balanced Salt Solution (HBSS) containing 5 mM EDTA, resuspended in flow cytometry buffer, blocked as described above then stained with anti-MHC II-PE (I-A/I-E; clone M5/114.15.2) or anti-CD86-PE (clone GL1) for 20 minutes on ice. Cells were gated on CD11b and the surface expression of Tim-3, MHC II or CD86 was determined by calculating mean fluorescence intensity using C6 Accuri system software (BD Bioscience, San Jose, CA, USA).

### Tim-3 inhibition

The contribution of Tim-3 in transducing the effects of Gal-9 was determined using mixed glial cultures stimulated with or without poly(I:C) in the presence of increasing concentrations of either a neutralizing antibody to Tim-3 (clone 8B.2C12) or isotype control antibody. To neutralize galectin-9/Tim-3 signaling by competitive ligation, mixed glial cultures were stimulated with or without poly(I:C) in the presence of increasing concentrations of soluble recombinant Tim-3 (Tim-3-Fc fusion protein). Levels of TNF in the supernatant were then determined by ELISA. All reagents were obtained from eBioscience (San Diego, CA, USA).

### Statistical analysis

All data are presented as means ± SE. To determine differences between experimental groups we employed two-tailed Student's *t*-tests for comparisons between two groups or analysis of variance (ANOVA) followed by Bonferroni's *post-hoc* test to compare multiple groups. All statistics were calculated using GraphPad Prism 4 (GraphPad Software, San Diego, CA, USA). In all cases differences were considered significant when *P* < 0.05.

## Results

### Astrocytes promote TNF production from microglia

Previously we found that microglia but not astrocytes secreted TNF following *in vitro* stimulation of mono- and mixed glial cultures with either LPS or poly(I:C) [41,43]. Interestingly however, while astrocyte mono-cultures failed to secrete TNF after either poly(I:C) or LPS stimulation, they drastically enhanced microglial TNF production when co-cultured with microglia (Figure 1A; Additional file 1: Figure S1A). This synergistic increase in cytokine production was dependent on the number of astrocytes, but not microglia in the co-culture as increasing the number of astrocytes cultured with microglia recapitulated the effect while increasing the number of microglia cultured with microglia failed to promote additional TNF production past a certain threshold (Figure 1B). The effect of astrocytes on microglia could be attributable to their ability to promote microglial proliferation and/or survival. However, immunocytochemical analysis as well as the measurement of lactate dehydrogenase release indicated that this was not the case (Additional file 1: Figure S1B and C). To determine if live astrocytes were necessary to enhance microglial TNF production, we fixed astrocytes with methanol prior to adding microglia (Additional file 2: Figure S2A). As observed in previous experiments, control astrocytes promoted TNF production from microglia following poly(I:C) stimulation (Additional file 2: Figure S2B). In contrast, astrocyte cultures that were fixed prior to stimulation with poly(I:C) failed to promote TNF production from microglia (Additional file 2: Figure S2A and B). Furthermore, we determined that this effect was contact dependent (Additional file 2: Figure S2C). Together, these results demonstrate that active communication between the two cell types is required for the enhanced microglial TNF production observed in the presence of astrocytes and indicate that an astrocyte-derived factor(s) is likely involved in the synergy.

### Stimulation of mixed glial cultures with the viral mimic poly(I:C) increases galectin-9

**Figure 2:**
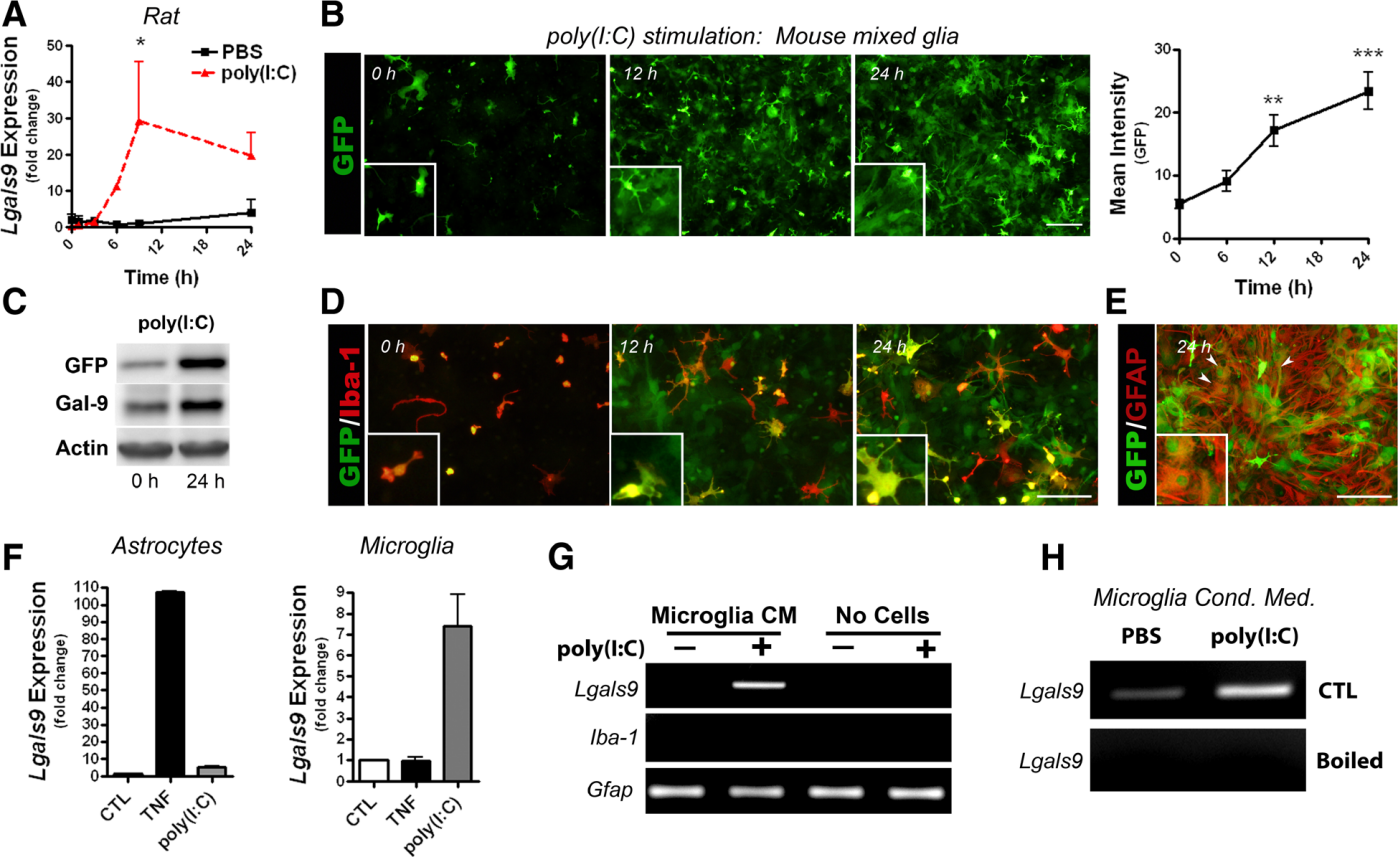
**Glia up-regulate galectin-9 following** ***in vitro*** **stimulation with poly(I:C). (A)** Fold change in galectin-9 mRNA following stimulation of rat mixed glia with vehicle control (PBS) or poly(I:C) (50 μg/ml) over time. Results are combined means ± SE from two independent experiments. **(B-E)** Mouse mixed glia from *Lgals9*:EGFP mice (n = 4) stimulated with poly(I:C) (50 μg/ml). Time-dependent increase in both galectin-9 promoter activation **(B)** and protein production **(C)** following stimulation. Galectin-9 localizes to both Iba-1^+^ microglia **(D)** and GFAP^+^ astrocytes **(E)**.** (F)** Relative *Lgals9* expression in astrocyte (left) or microglia (right) monocultures following treatment with either recombinant TNF (5 ng/ml) or poly(I:C) (50 μg/ml). Results are means ± SE of duplicate samples and are representative of three independent experiments. **(G)** **(H)** Conditioned media from poly(I:C)-activated microglia is sufficient to induce *Lgals9* expression in rat astrocyte cultures. Media obtained from microglia treated with vehicle (PBS) or poly(I:C) (50 μg/ml) for 24 hours was used to treat astrocytes for 7 hours and *Lgals9* up-regulation determined by RT-PCR (left). Microglial conditioned media was heated to 100°C before adding to astrocytes (right). Results are representative of two to four independent experiments. *P < 0.05, **P < 0.01, ***P < 0.001.

Human and rodent astrocytes up-regulate galectin-9 following stimulation with various proinflammatory cytokines, including IL-1β and TNF [36,45], both of which are released from activated microglia. Furthermore, recombinant galectin-9 is reported to enhance innate immune responses of dendritic cells [34] as well as promotes dendritic cell maturation [33]. Therefore, we first examined if poly(I:C) induces galectin-9 expression in mixed glia. Stimulation of both rat (Figure 2A) and mouse (Figure 2B) mixed glia with poly(I:C) resulted in up-regulation of *Lgals9* in a time-dependent fashion. Utilizing mixed glia from galectin-9 EGFP mice (*Lgals9*:EGFP) that contain EGFP expression cassette after galectin-9 promoter, we found that poly(I:C) time-dependently increased galectin-9 expression as determined by increased fluorescence intensity of GFP (Figure 2B) as well as western blot analysis (Figure 2C). Similar responses were observed following LPS stimulation (Additional file 3: Figure S3A and B). In line with our previous results [36], microglia constitutively expressed *Lgals9* (Figure 2B). However, in mixed glial cultures poly(I:C) time-dependently induced EGFP expression in cells morphologically resembled astrocytes (Figure 2D), a finding that was further confirmed by immunocytochemical analysis, which revealed co-localization of GFP^+^ cells with GFAP^+^ astrocytes (Figure 2E). Interestingly, microglia but not astrocyte monocultures challenged with poly(I:C) or LPS induced *Lgals9* expression (Figure 2F*;* Additional file 3: Figure S3C). As before [36], stimulation of astrocytes but not microglia monocultures with TNF induced *Lgals9* expression (Figure 2F), indicating that stimulation of microglia with poly(I:C) up-regulates galectin-9 whereas microglial-derived cytokines such as TNF causes galectin-9 up-regulation in astrocytes. Indeed, conditioned media from poly(I:C) stimulated microglia, but not media containing poly(I:C), increased *Lgals9* expression in astrocytes (Figure 2G). This effect was abolished if the conditioned media was boiled prior to its addition to astrocytes (Figure 2H), demonstrating that the microglial factors that induce *Lgals9* transcription are heat sensitive.

**Figure 3:**
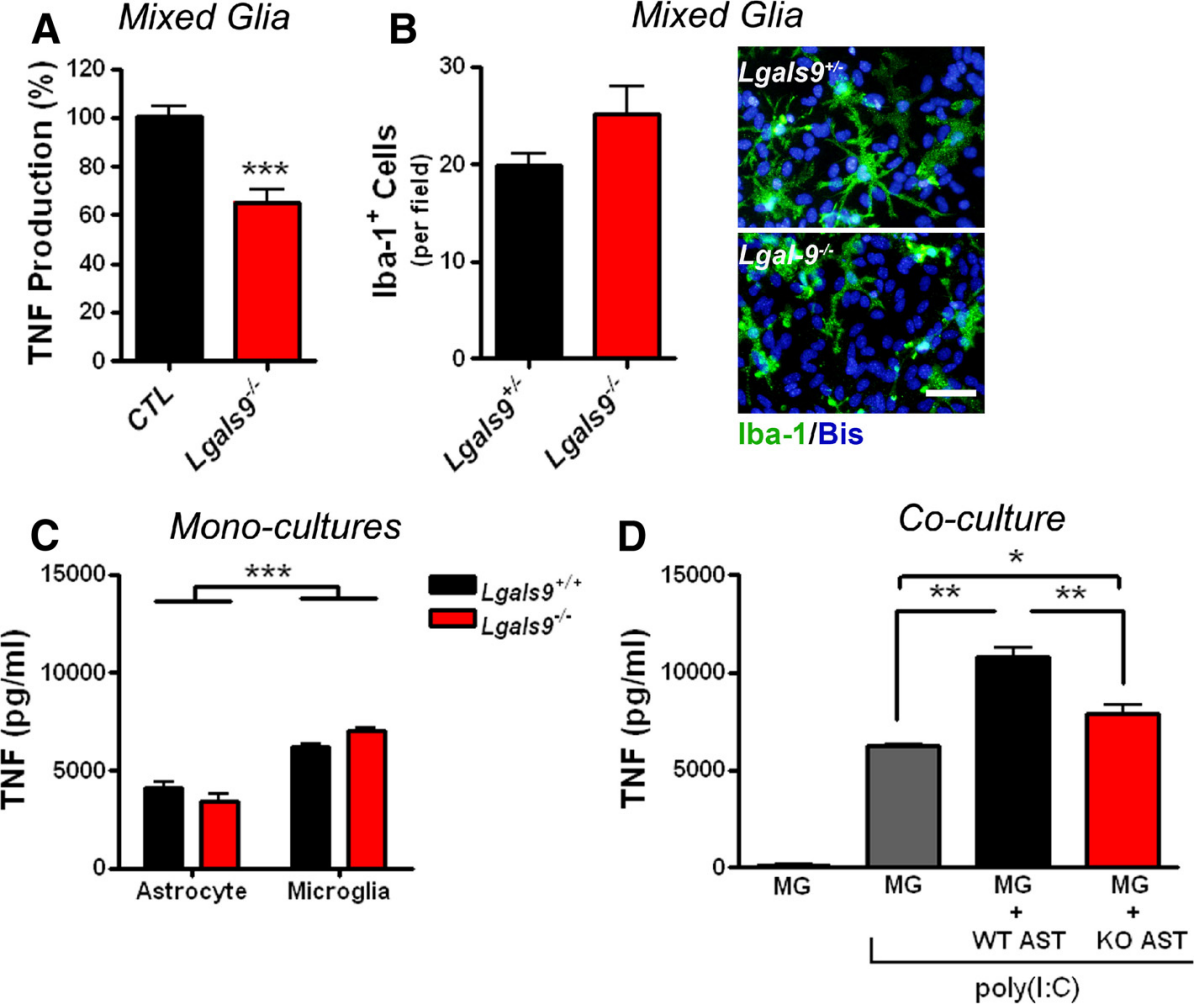
**Astrocyte-derived galectin-9 promotes microglial TNF production.** Mono-, co- or mixed glial cultures obtained from littermate control (*Lgals9* ^*+/+*^ or *Lgals9* ^*+/−*^) or *Lgals9* ^*−/−*^ mice were stimulated with poly(I:C) (25 μg/ml) for 24 hours and TNF measured from the supernatants by ELISA. **(A-B)** Reduction in supernatant levels of TNF obtained from mixed glia **(A)** despite similar numbers of Iba-1^+^ microglia across genotypes **(B)**. Results in **(A)** are combined means ± SE of 5 independent experiments (*CTL n* = 19; *Lgals9* ^*−/−*^ *n* = 12); results in **(B)** are combined means ± SE of glial cultures depicted in **(A)** chosen at random (*Lgals-9* ^*+/−*^ *n* = 3; *Lgals9* ^*−/−*^ *n* = 3). Scale bar, 50 μm. **(C)** TNF levels from poly(I:C) activated astrocyte and microglial monocultures derived from *Lgals-9* ^*+/+*^ or *Lgals9* ^*−/−*^ mice. Results are means ± SE of quadruplicate samples and are representative of two independent experiments. **(D)** Supernatant levels of TNF following poly(I:C) stimulation of microglial monocultures compared to co-cultures containing *Lgals-9* ^*+/+*^ (wild-type (WT); *black*) or *Lgals9* ^*−/−*^ (knockout (KO); *red*) astrocytes. Results are means ± SE of quadruplicate samples and are representative of four independent experiments. *P < 0.05, **P < 0.01, ***P < 0.001.

### Astrocyte-derived galectin-9 promotes microglial TNF secretion

To determine if galectin-9 was responsible for the synergic response from astrocytes, we stimulated control (*Lgals9*^*+/+*^ or *Lgals9*^*+/−*^) and *Lgals9*^−/−^ mixed glial cultures with poly(I:C) and measured TNF secretion. There were no differences in TNF production from *Lgals9*^*+/+*^ cultures compared to *Lgals9*^*+/−*^ cultures following poly(I:C) stimulation (not shown). However, mixed glia derived from *Lgals9*^*−/−*^ mice produced approximately 35% less TNF upon activation than control cultures (Figure 3A). The number of microglial cells in these cultures did not differ between genotypes (Figure 3B), thereby excluding the possibility that the decrease in TNF production was attributable to culture variation. Since microglia constitutively express galectin-9 (Figure 2D and [36]), we tested the possibility that a lack of microglial galectin-9 was responsible for the reduction in TNF. However, primary microglia isolated from *Lgals9*^*+/+*^ and *Lgals9*^*−/−*^ mice produced similar amounts of TNF after poly(I:C) stimulation (Figure 3C). Likewise, production of TNF in enriched astrocyte cultures, which is attributable to residual contaminating microglia [43] was similar between genotypes (Figure 3C). To confirm that astrocyte-derived galectin-9 was needed to synergize microglia TNF production, we examined the effect of stimulation on microglia alone or co-cultured with either *Lgals9*^*+/+*^ or *Lgals9*^*−/−*^ astrocytes. Stimulation of microglia co-cultured with *Lgals9*^*+/+*^ astrocytes produced more TNF than either microglia alone or microglia co-cultured with *Lgals9*^*−/−*^ astrocytes, demonstrating that astrocyte-derived galectin-9 promotes TNF production by microglia (Figure 3D).

### Recombinant galectin-9 is sufficient to promote proinflammatory responses from microglia

**Figure 4:**
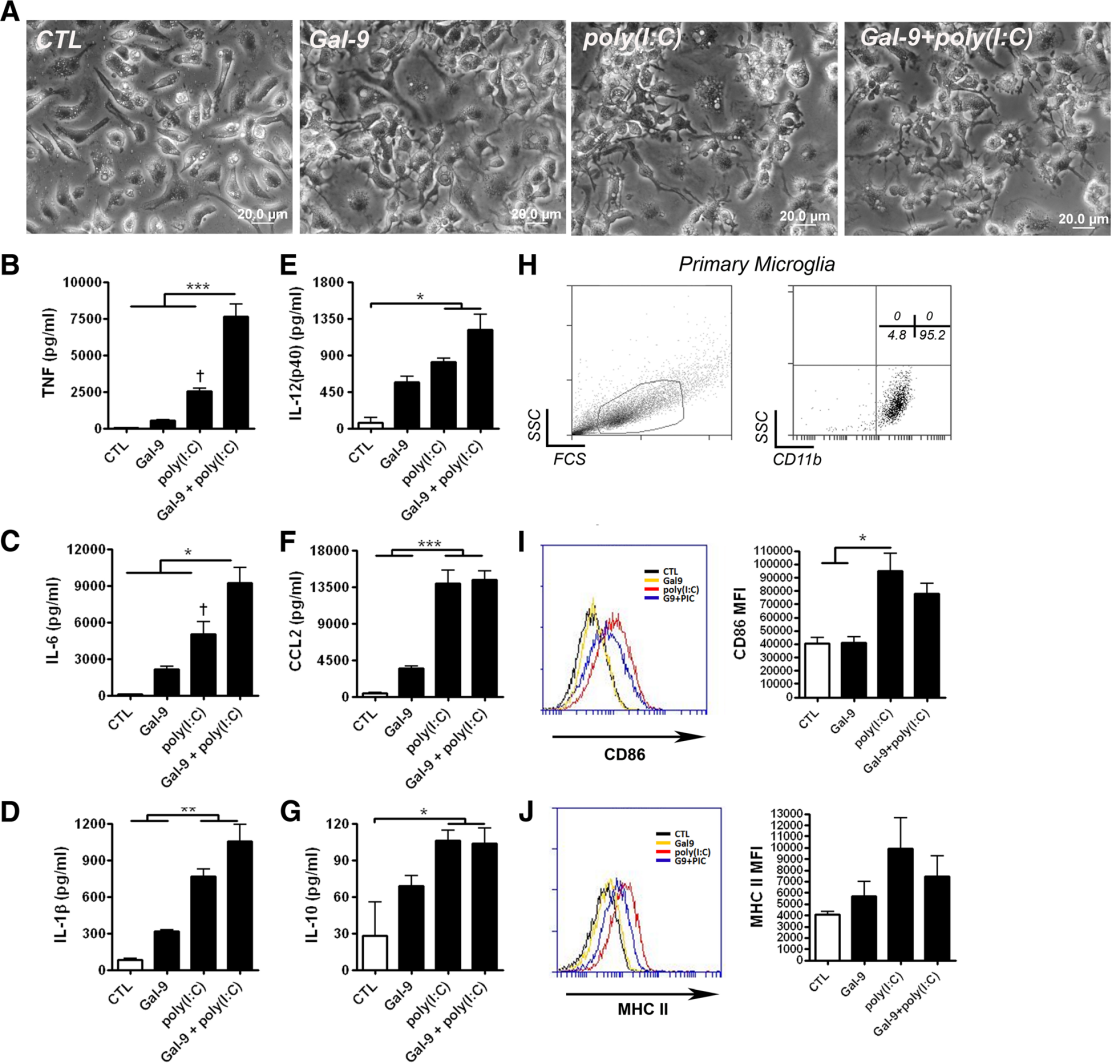
**Recombinant galectin-9 synergizes microglial TNF and IL-6 production. (A)** Primary mouse microglia were unstimulated (CTL) or stimulated with recombinant mouse galectin-9 (2 μg/ml), poly(I:C) (25 μg/ml), or galectin-9 and poly(I:C) together for 24 hours and cell morphology examined by microscopy **(A)**. **(B-F)** Following the above treatments chemokines and cytokines were simultaneously measured from supernatants by Bioplex assay. Levels of TNF **(B)**, IL-6 **(C)**, IL-1β **(D)**, IL-12(p40) **(E)**, CCL2 **(F)** and IL-10 **(G)** are shown. Results are means ± SE of triplicate samples and are representative of three independent experiments. *P < 0.05, **P < 0.01, ***P < 0.001; †*P* < 0.05 versus control. **(H-J)** Primary mouse microglia were stimulated as above for 24 hours, gated on CD11b **(H)** and the surface expression of CD86 **(I)** and MHC II **(J)** determined by flow cytometry. Representative histograms (left) as well as combined mean fluorescence intensity values from three independent experiments (right) are shown. Results are combined means ± SE of three independent experiments.

Treatment of primary microglia with recombinant galectin-9, poly(I:C), or both for 24 hours changed the cell morphology (Figure 4A). To further characterize the effects of galectin-9 on the innate immune response from microglia, we examined TNF, IL-6, IL-1β, CCL2, IL-10, and IL-12(p40) secretion after poly(I:C) stimulation. Although stimulation with exogenously added recombinant galectin-9 appeared to increase the production of most cytokines over controls, this effect did not reach statistical significance (one-way ANOVA). In contrast, poly(I:C) stimulation increased the secretion of all measured cytokines (Figure 4B, C, D, E, F and G). Interestingly, microglial TNF was significantly increased in poly(I:C) plus galectin-9 treated cultures when compared to cultures treated with only poly(I:C) (Figure 4B). While, galectin-9 co-treatment with poly(I:C) marginally enhanced IL-6 production when compared to poly(I:C) treatment alone, the effect was not as robust as that observed for TNF (Figure 4C). Importantly, the effects of recombinant galectin-9 were unlikely to be attributable to residual endotoxin resulting from the purification of recombinant galectin-9 as the treatment of primary microglia derived from *Tlr4* null mice with galectin-9 also up-regulated TNF (Additional file 4: Figure S4).

To test if galectin-9 alters the antigen presenting capacity of microglia we cultured microglia in the presence of galectin-9, poly(I:C), or both for 24 hours and analyzed surface expression of MHC II and the co-stimulatory molecule CD86 on CD11b^+^ gated cells by flow cytometry (Figure 4H-J). While poly(I:C) up-regulated the surface expression of CD86 (Figure 4I) on microglia, recombinant galectin-9 did not synergize this response. Collectively, these data demonstrate the ability of exogenous galectin-9 to synergize poly(I:C) mediated TNF and to a lesser extent IL-6 production in microglia.

### Microglial Tim-3 expression does not account for the effects of galectin-9

**Figure 5:**
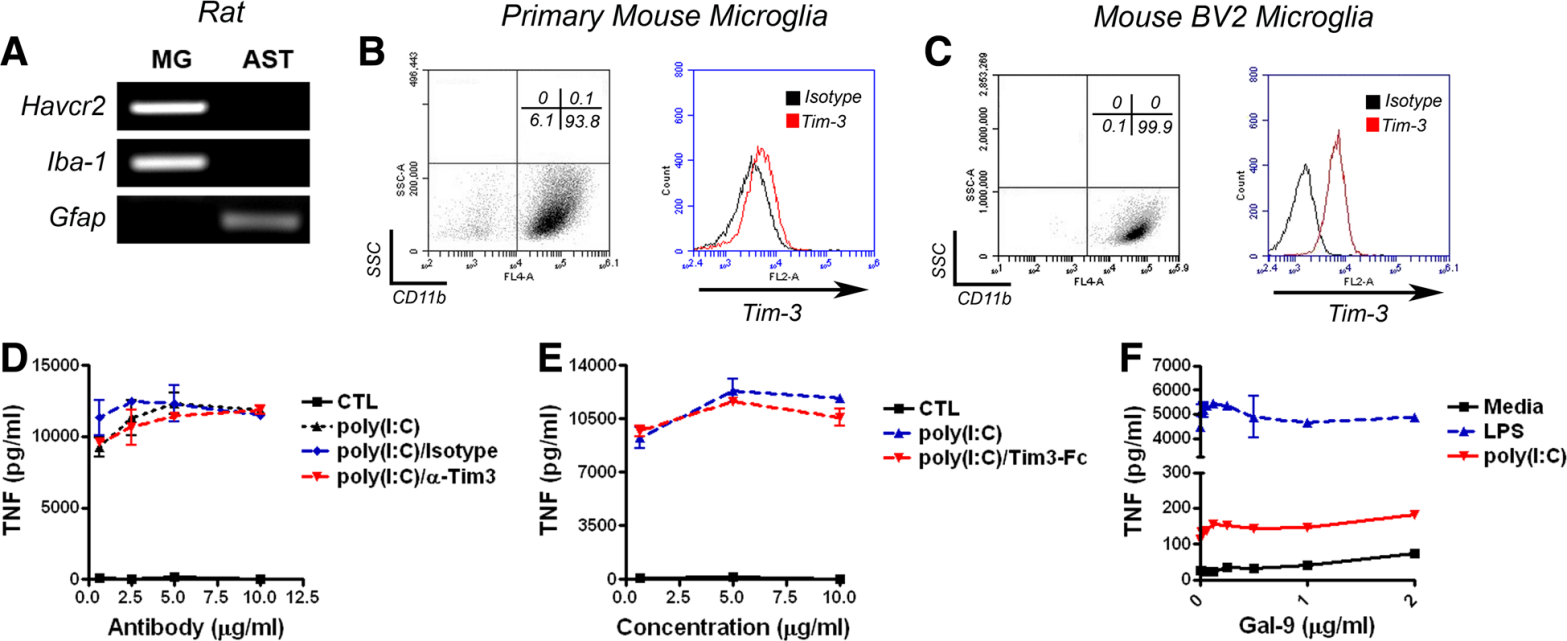
**Tim-3 is not required for the effects of galectin-9 on microglial cytokine production. (A)** RNA was extracted from rat microglia or astrocytes. Expression of *Havcr3* (top), the microglia marker *Iba-1* (middle), and the astrocyte marker *Gfap* (bottom) were examined by RT-PCR. **(B-C)** Surface expression of Tim-3 was examined on CD11b^+^ primary mouse microglia (**B**; left) and BV2 cells (**C**; right) by flow cytometry. Results are representative of three independent experiments for each cell type. **(D-E)** The effect of Tim-3 neutralization on TNF production in mouse mixed glial cultures. **(D)** Cultures were stimulated with poly(I:C) for 24 hours in the presence of increasing concentrations of a neutralizing antibody to Tim-3 (8B.2C12) or an isotype control (rat IgG1). **(E)** Cultures were stimulated with poly(I:C) and increasing concentrations of soluble Tim-3. Results are means ± SE of duplicate samples and are representative of three independent experiments for each experiment. **(F)** Mouse BV2 cells were challenged with vehicle, LPS (100 ng/ml) or poly(I:C) (25 μg/ml) in the presence of increasing concentrations of recombinant mouse galectin-9 (2 μg/ml) and TNF levels measured after 24 hours. Results are means ± SE of duplicate samples and are representative of two independent experiments.

Galectin-9 has been shown to increase TNF production from dendritic cells after LPS stimulation in a manner dependent on Tim-3 [34]. As such, we questioned if the effects of galectin-9 on microglia TNF production in mixed glial cultures (Figure 3A) were mediated by Tim-3 signaling. To begin, we first examined if Tim-3 was expressed on microglia. Rat microglia but not astrocytes constitutively expressed *Havcr2* (the gene encoding Tim-3) mRNA (Figure 5A). Similarly, flow cytometry analysis of primary mouse microglia revealed low surface expression levels of Tim-3, whereas the mouse microglia cell line (BV2) exhibited much higher level of surface Tim-3 (Figure 5B and C). Consistent with the low Tim-3 surface expression on microglia, poly(I:C) stimulation of mixed glia in the presence of increasing concentrations of a neutralizing antibody to Tim-3 (clone 8B.2C12) did not suppress the amount of TNF produced when compared to cultures co-treated with vehicle (PBS) or an isotype control antibody (Figure 5D). Furthermore, addition of excess soluble Tim-3 (Tim-3-Fc fusion protein) also failed to alter poly(I:C)-induced TNF secretion, indicating that galectin-9 is not sequestered by Tim-3-Fc or has a higher affinity/avidity for a different glycoconjugate through which it mediates its effects (Figure 5E). Finally, despite that BV2 cells abundantly express surface Tim-3, stimulation of this BV2 microglial cell line with recombinant galectin-9 did not increase TNF production when concurrently administered with either poly(I:C) or LPS (Figure 5F). Together these data strongly suggest that galectin-9 is capable of acting in a Tim-3 independent fashion to promote TNF production from activated microglia.

## Discussion

In the current study we demonstrate that astrocytes enhance TNF production from activated microglia and that this effect is primarily dependent on galectin-9. We show that stimulation of glial cultures with the viral mimic poly(I:C) or the Gram-negative bacterial product LPS results in galectin-9 up-regulation in both microglia and astrocytes via different signaling pathways. In line with our finding that galectin-9 promotes microglial responses, we demonstrate that recombinant galectin-9 synergizes with poly(I:C) to increase TNF and IL-6 secretion from cultured microglia. Finally, our data suggest that the synergistic action of galectin-9 and poly(I:C) on microglial TNF production is Tim-3 independent and thus is likely to be transduced through an additional, yet to be identified, glycoconjugate.

While galectin-9 is minimally expressed in the unchallenged brain, its expression has been reported to increase following acute infection with herpes simplex virus [46] and during pneumococcal meningitis [37] indicating that CNS infection likely promotes galectin-9 up-regulation. Moreover, our current and previous data provide some evidence regarding the mechanism of up-regulation for each distinct cell type within the CNS following infection. Similar to that reported for human umbilical vein endothelial cells [47], we found galectin-9 also is constitutively expressed in cerebrovascular endothelial cells (Steelman and Li personal observations). Additionally, we found galectin-9 expression localized to quiescent resident microglia in naïve animals (Steelman and Li personal observations), a finding that complements *in vitro* data from previous experiments [36]. As in HUVEC [48,49] and mesenchymal stromal cells [50], we report here that galectin-9 expression can be induced in microglia following poly(I:C) stimulation. However, despite astrocytes possessing poly(I:C) receptors and being capable of producing CCL2/5 in response to poly(I:C) stimulation [43], they themselves did not directly up-regulate galectin-9 (Figure 2F) or produce TNF [43] upon poly(I:C) stimulation. Moreover, astrocytes neither bind to LPS nor express TLR4 [41,51,52] and did not increase *Lgals9* expression following LPS stimulation (Additional file 3: Figure S3C). Together these findings may suggest that epigenetic regulation of inflammatory genes differ between microglia and astrocytes, a hypothesis that has great implications but has yet to be thoroughly tested. Nevertheless, it is likely that increased galectin-9 expression in astrocytes is dependent on proinflammatory cytokines from the surrounding milieu. The sufficiency of conditioned media from poly(I:C) activated microglia to up-regulate astroglial galectin-9 expression supports this hypothesis. As such, following acute viral infection microglia and endothelial cells are likely to increase galectin-9 directly in response to pattern recognition receptor activation whereas up-regulation in astrocytes depends on proinflammatory cytokines present in the surrounding tissue.

Understanding the physiological consequence of increased galectin-9 expression within the CNS during encephalitis brought on by central or peripheral infection or autoimmunity is an important step towards understanding its therapeutic potential or pitfalls under various causes of chronic neuroinflammation. At present most previous studies have addressed the effects of galectin-9 up-regulation with regard to its function on adaptive immunity. Fewer studies have addressed the effects of galectin-9 on innate immunity with some conflicting results. For instance, it has been reported that galectin-9 can promote innate immune responses from antigen presenting cells [34] and that galectin-9 can promote dendritic cell maturation from monocyte-derived macrophages [53]. Moreover, activation of Tim-3 by galectin-9 is reported to aid in the clearance of mycobacterial tuberculosis which is mediated by increased IL-1β release from monocytes [54,55]. However, siRNA mediated abolition of Tim-3 in monocytes has been shown to cause increased production of IL-12(p70), an effect that was attributable to the cis association of galectin-9 with Tim-3 [56,57].

To our knowledge, the current study represents the first to demonstrate a role for galectin-9 in glial activation. Recently, Lee and Goverman have investigated the role of Tim-3 in antigen presenting cell function as it relates to EAE [58]. In these experiments they demonstrated that microglia isolated from both the quiescent and inflamed CNS express Tim-3. However, microglial Tim-3 did not alter the pathogenesis of EAE as bone marrow chimeric mice generated from Tim-3 null mice reconstituted with wild-type bone marrow did not differ in disease severity when compared to controls [58]. Furthermore, they did not find evidence to support a role for Tim-3 in modulating proinflammatory cytokine release or antigen presenting cell function from isolated dendritic cells [58]. In line with these data, the results from the current study suggest that galectin-9 can promote inflammatory cytokine production, but does so in a manner that is not, to say the least, entirely dependent on Tim-3 signaling. Some of these seemingly conflicting results may be partially explained by the promiscuous nature of galectin-glycoprotein interactions. As galectins have an affinity for specific glycan moieties on glycoconjugates they would be expected to have multiple binding partners. Indeed, in addition to Tim-3 [59] galectin-9 has also been shown to bind CD40 [60], CD44 [61,62], protein disulfide isomerase [62], ICAM3, PECAM1, Integrin β1, Integrin β2, Integrin αL, CD2, CD6, CD26, CD45, CD48, CD107a, and CD148 [61]. Finally, the glycosylation status of proteins greatly influences galectin avidity [30,63]. Therefore, variation experimental culture conditions could potentially account for some discrepancies.

## Conclusion

We have demonstrated that astrocyte-derived galectin-9 potentiates TNF and to a lesser extent IL-6 production from microglia *in vitro* in a Tim-3 independent manner. Galectin-9 expression is increased in microglia following TLR ligation. However, its expression in astrocytes is likely dependent on microglia-derived cytokine production, at least *in vitro*. Initially, this physiological response may function to promote pathogen clearance from the CNS. However, the pathophysiological outcome of galectin-9 up-regulation within the CNS is incompletely understood. Whether glia-derived galectin-9 promotes or suppresses neuroinflammatory diseases is currently the subject of ongoing investigation.

## Additional files

Additional file 1: Figure S1. Enhanced TNF production in microglia/astrocyte co-cultures is observed following LPS stimulation. **(A)** TNF levels from supernatants of mono- and co-cultures of astrocytes and microglia following stimulation with or without LPS (100 ng/ml) for 24 hours. Results are combined means ± SE of three independent experiments. **(B)** The number of microglia in each condition was determined by counting Iba-1^+^ cells in cultured treated with or without poly(I:C) (50 μg/ml) for 24 hours. The results are mean ± SE of 5 to 7 20x fields from a single experiment and are representative of 2 independent experiments. ***P* < 0.01, ****P* < 0.001. **(C)** Lactate dehydrogenase (LDH) levels in culture supernatants from experiment **(B)**. Additional file 2: Figure S2. Live astrocytes promote microglia TNF production in a contact-dependent manner. **(A)** Schematic illustration of the experimental design in **(B)**. Astrocytes were plated at 5 × 10^4^ cells per well in a 96-well plate. After 22 hours the cells were incubated with either PBS (left) or ice-cold methanol (right) for 10 minutes at RT. After washing twice with warm media, 5 × 10^4^ microglia were added to each well. The following day the cells were treated with poly(I:C) (50 μg/ml) for 0, 4, 8 and 24 hours. **(B)** ELISA results showing TNF secretion from microglia monocultures, microglia co-cultured with live astrocytes, microglia co-cultured with methanol-fixed astrocytes, or astrocyte monocultures following poly(I:C) stimulation. Results are means ± SE from triplicate wells. **(C)** Schematic illustration of the no-contact experimental design whereby microglia and astrocytes were cultured together but physically separated. The illustration is a reproduction from a similar illustration published previously [41]. **(D)** TNF production from microglia cultured alone or in the presence of astrocytes with (black bars) or without (white bars) contact following poly(I:C) (50 μg/ml) for 24 hours. Results are means ± SE from triplicate measurements of two separate wells per condition and are representative of three independent experiments. ***P* < 0.01, ****P* < 0.001. Additional file 3: Figure S3. LPS up-regulates galectin-9. **(A and B)** Mixed glia from *Lgals9:EGFP* mice were stimulated with LPS (100 ng/ml) and time-dependent increase in galectin-9 promoter activation was evaluated. **(B)** is the mean fluorescence intensity from duplicates over time. Scale bar, 100 μm. **(C)** The effect of TNF (5 ng/ml) and LPS (100 ng/ml) on *Lgals9* expression in rat astrocytes (left) or microglia (right) monocultures as determined by RT-qPCR. Results are means ± SE of triplicate samples and are representative of two independent experiments. Additional file 4: Figure S4. The effects of galectin-9 are not attributable to residual endotoxin. Microglia were isolated from wild-type C57BL/6 mice (WT) or toll-like receptor mutant mice (*Tlr4* ^*−/−*^) and plated into 96-well plates at 5 × 10^4^ cells per well. Microglia from WT (*left*) or *Tlr4* ^*−/−*^ (*right*) mice were treated with or without recombinant galectin-9 (2 μg/ml) for 24 hours and TNF levels determined.

## Abbreviations

CNS: central nervous system
EAE: experimental autoimmune encephalomyelitis
IL-6: interleukin-6
LPS: lipopolysaccharide
Tim-3: glycoprotein T-cell immunoglobulin and mucin-domain containing protein 3
TLR: toll-like receptor
TNF: tumor necrosis factor
