# Supplementary figures and images for: Astrocyte galectin-9 potentiates microglial TNF secretion
Source: J Neuroinflammation. 2014 Aug 27;11:144. doi: 10.1186/s12974-014-0144-0 (PMC4158089; doi:10.1186/s12974-014-0144-0)

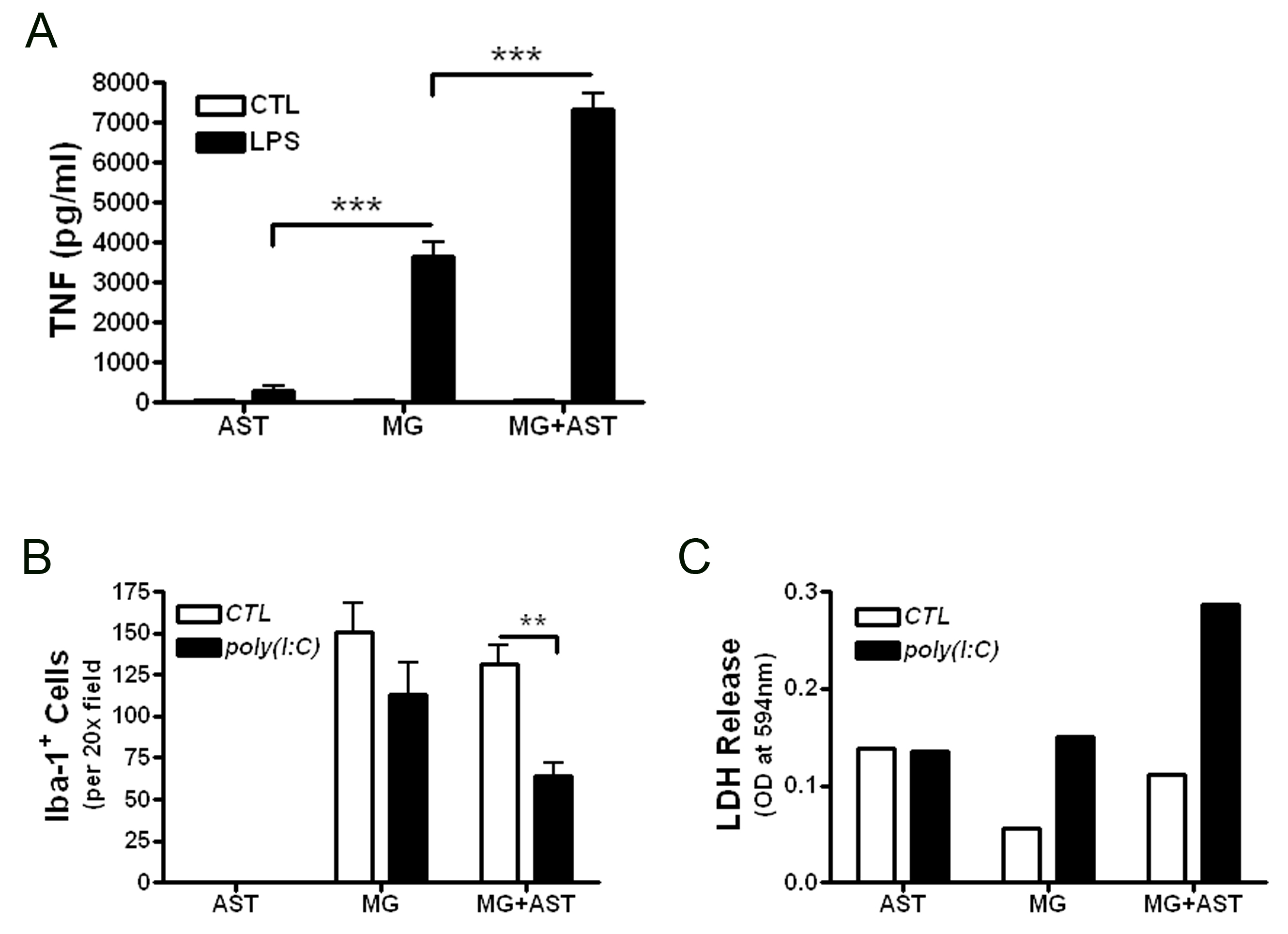

Supplement: Additional file 1: Figure S1. — Enhanced TNF production in microglia/astrocyte co-cultures is observed following LPS stimulation. (A) TNF levels from supernatants of mono- and co-cultures of astrocytes and microglia following stimulation with or without LPS (100 ng/ml) for 24 hours. Results are combined means ± SE of three independent experiments. (B) The number of microglia in each condition was determined by counting Iba-1+ cells in cultured treated with or without poly(I:C) (50 μg/ml) for 24 hours. The results are mean ± SE of 5 to 7 20x fields from a single experiment and are representative of 2 independent experiments. **P < 0.01, ***P < 0.001. (C) Lactate dehydrogenase (LDH) levels in culture supernatants from experiment (B). [file 12974_2014_144_MOESM1_ESM.jpg]

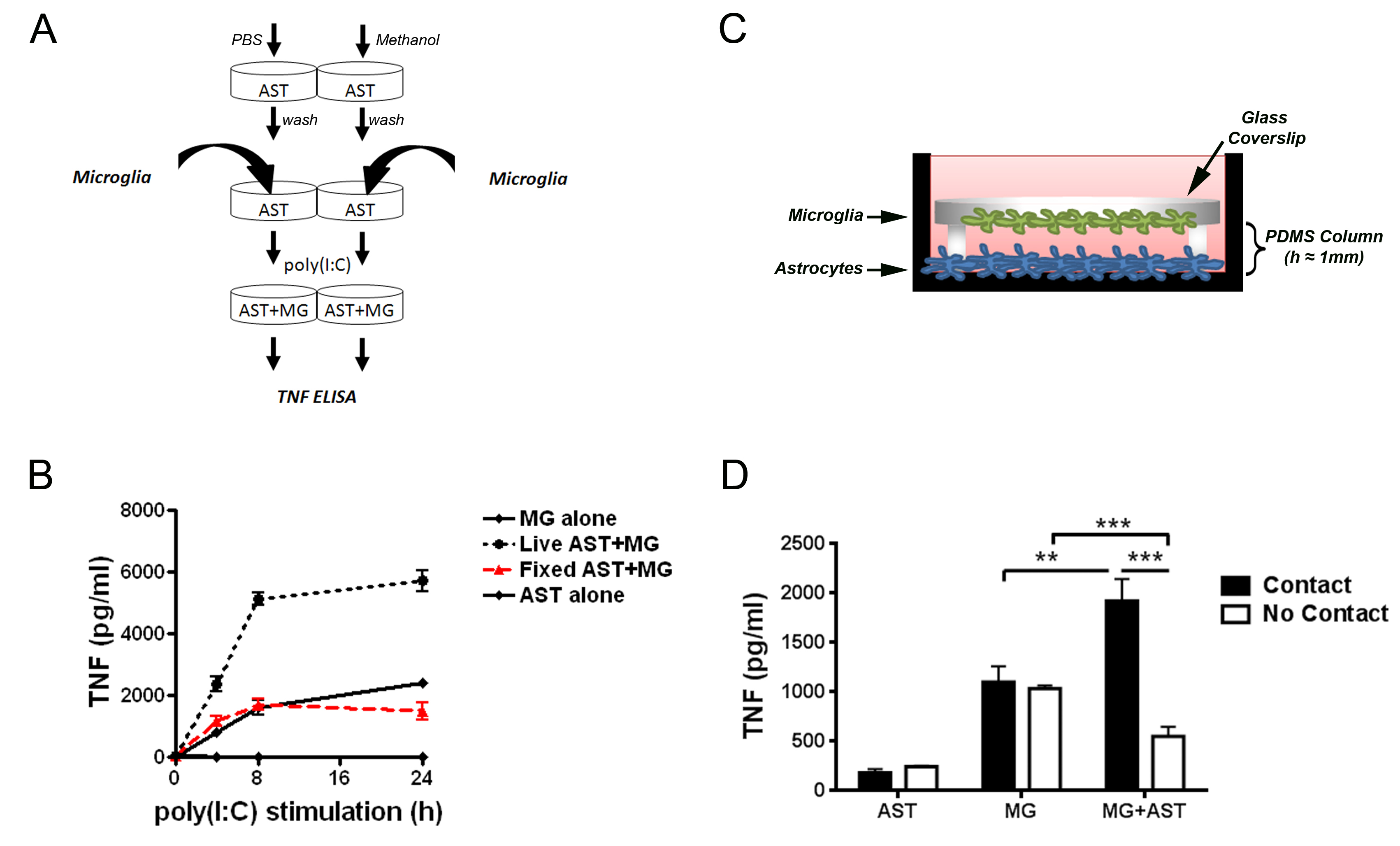

Supplement: Additional file 2: Figure S2. — Live astrocytes promote microglia TNF production in a contact-dependent manner. (A) Schematic illustration of the experimental design in (B). Astrocytes were plated at 5 × 10^4^ cells per well in a 96-well plate. After 22 hours the cells were incubated with either PBS (left) or ice-cold methanol (right) for 10 minutes at RT. After washing twice with warm media, 5 × 10^4^ microglia were added to each well. The following day the cells were treated with poly(I:C) (50 μg/ml) for 0, 4, 8 and 24 hours. (B) ELISA results showing TNF secretion from microglia monocultures, microglia co-cultured with live astrocytes, microglia co-cultured with methanol-fixed astrocytes, or astrocyte monocultures following poly(I:C) stimulation. Results are means ± SE from triplicate wells. (C) Schematic illustration of the no-contact experimental design whereby microglia and astrocytes were cultured together but physically separated. The illustration is a reproduction from a similar illustration published previously [41]. (D) TNF production from microglia cultured alone or in the presence of astrocytes with (black bars) or without (white bars) contact following poly(I:C) (50 μg/ml) for 24 hours. Results are means ± SE from triplicate measurements of two separate wells per condition and are representative of three independent experiments. **P < 0.01, ***P < 0.001. [file 12974_2014_144_MOESM2_ESM.jpg]

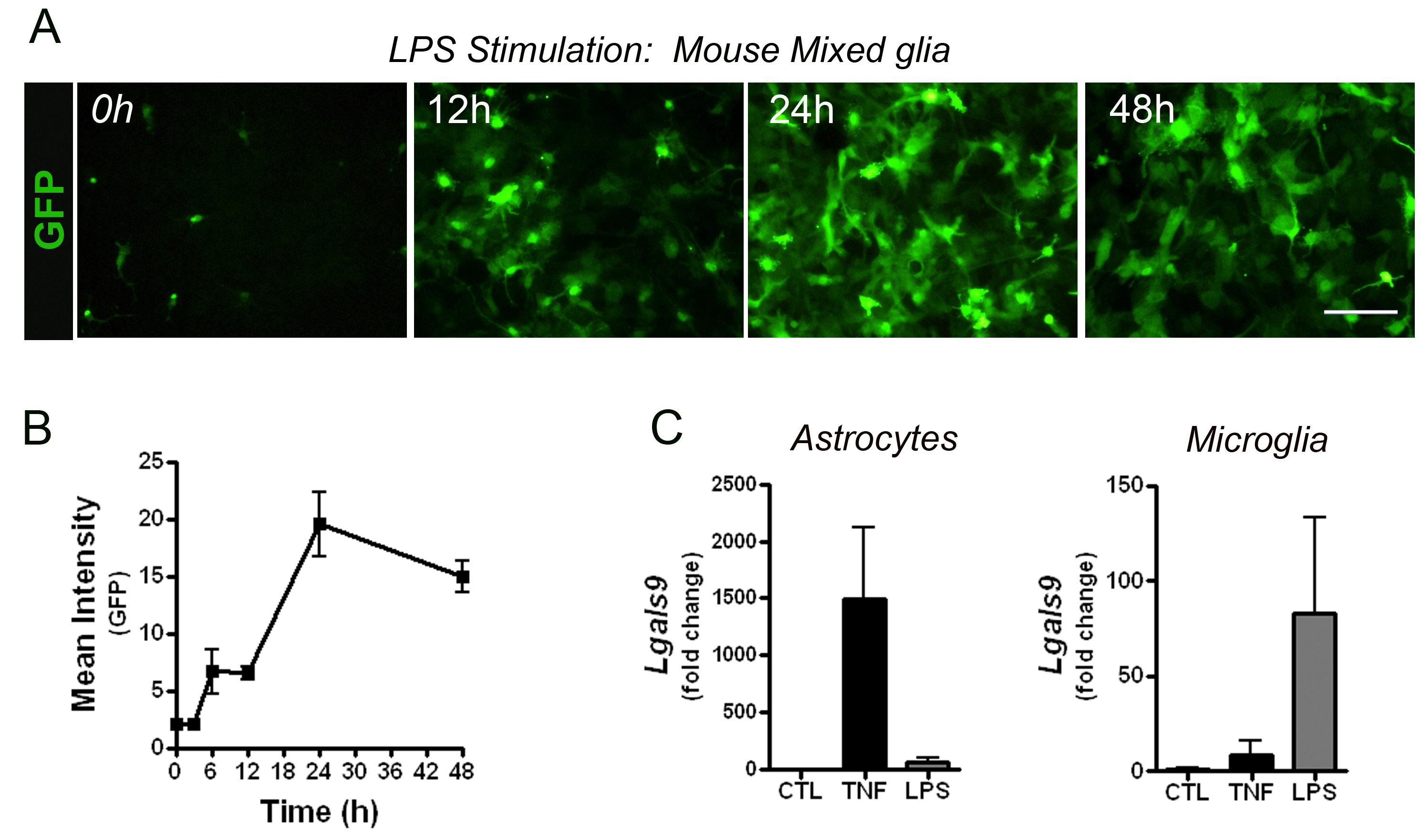

Supplement: Additional file 3: Figure S3. — LPS up-regulates galectin-9. (A and B) Mixed glia from Lgals9:EGFP mice were stimulated with LPS (100 ng/ml) and time-dependent increase in galectin-9 promoter activation was evaluated. (B) is the mean fluorescence intensity from duplicates over time. Scale bar, 100 μm. (C) The effect of TNF (5 ng/ml) and LPS (100 ng/ml) on Lgals9 expression in rat astrocytes (left) or microglia (right) monocultures as determined by RT-qPCR. Results are means ± SE of triplicate samples and are representative of two independent experiments. [file 12974_2014_144_MOESM3_ESM.jpg]

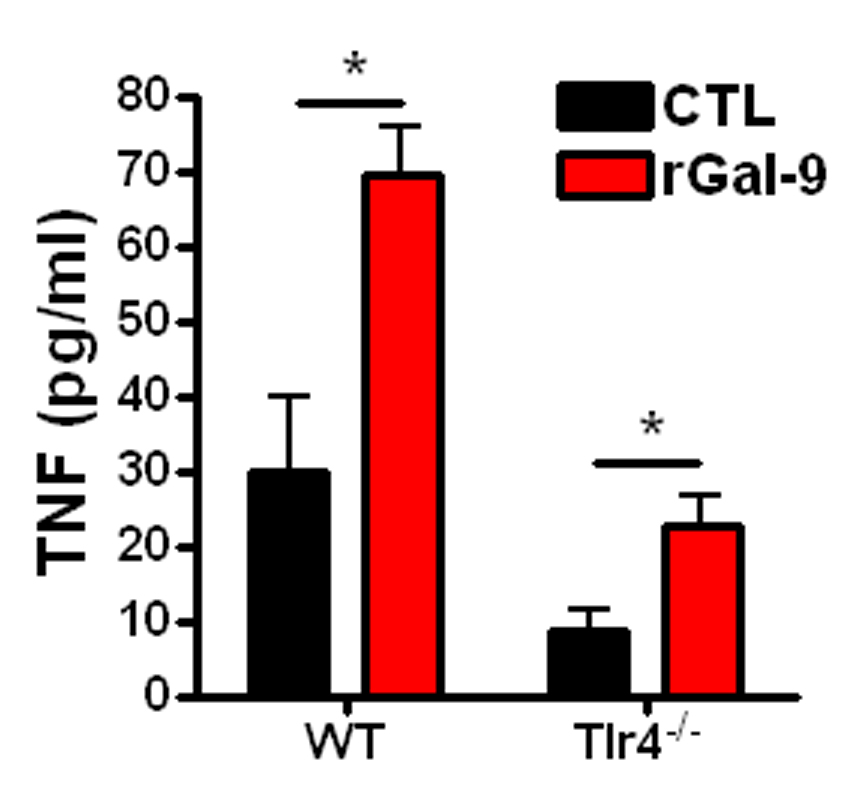

Supplement: Additional file 4: Figure S4. — The effects of galectin-9 are not attributable to residual endotoxin. Microglia were isolated from wild-type C57BL/6 mice (WT) or toll-like receptor mutant mice (Tlr4 −/−) and plated into 96-well plates at 5 × 10^4^ cells per well. Microglia from WT (left) or Tlr4 −/− (right) mice were treated with or without recombinant galectin-9 (2 μg/ml) for 24 hours and TNF levels determined. [file 12974_2014_144_MOESM4_ESM.jpg]
